# Independent Predictors of Mortality in COVID-19 Myocardial Injury: The Role of Troponin Levels, GRACE Score, SOFA Score, and TIMI Score

**DOI:** 10.7759/cureus.32082

**Published:** 2022-11-30

**Authors:** Kavin Raj, Pranav Mahajan, Abi Watts, Surya Aedma, Jemin Aby Jose, Keerthana Jyotheeswara Pillai, Adam Rizkalla, Suyansh Sharma, Riddhi Upadhyay, Swati Dhobale, Talha Bin Farooq, Rukhsaar Khanam, Keval V Patel, Randolph Martin

**Affiliations:** 1 Cardiology, University of California, Riverside, San Bernardino, USA; 2 Internal Medicine, Carle Foundation Hospital, Urbana, USA; 3 Internal Medicine, Saint Peter’s University Hospital, New Brunswick, USA; 4 Internal Medicine, The University of Arizona, Tucson, USA; 5 Internal Medicine, Government Kilpauk Medical College, Chennai, IND; 6 Surgery, Mount Sinai Medical Center, New York, USA; 7 Critical Care, University of Miami Miller School of Medicine, Jackson Memorial Hospital, Miami, USA; 8 Internal Medicine, Hartford Hospital, Hartford, USA; 9 Cardiology, Rutgers-Robert Wood Johnson University Hospital, New Brunswick, USA; 10 Cardiology, Saint Peter’s University Hospital, New Brunswick, USA; 11 Cardiology, Carle Foundation Hospital, Urbana, USA

**Keywords:** covid-19, timi score, grace score, sofa score, myocardial infarction type 2, cardiac troponin

## Abstract

Background

Coronavirus disease 2019 (COVID-19) infection is associated with troponin elevation, which is associated with increased mortality. However, it is not clear if troponin elevation is independently linked to increased mortality in COVID-19 patients. Although there is considerable literature on risk factors for mortality in COVID-19-associated myocardial injury, the Global Registry of Acute Coronary Events (GRACE), Thrombolysis in Myocardial Infarction (TIMI), and Sequential Organ Failure Assessment (SOFA) scores have not been studied in COVID-19-related myocardial injury. This data is important in risk-stratifying COVID-19 myocardial injury patients.

Methodology

Of the 1,500 COVID-19 patients admitted to our hospitals, 217 patients who had troponin levels measured were included. Key variables were collected manually, and univariate and multivariate cox regression analysis was done to determine the predictors of mortality in COVID-19-associated myocardial injury. The differences in clinical profiles and outcomes of COVID-19 patients with and without troponin elevation were compared.

Results

Mortality was 26.5% in the normal troponin group and 54.6% in the elevated troponin group. Patients with elevated troponins had increased frequency of hypotension (p = 0.01), oxygen support (p < 0.01), low absolute lymphocyte (p < 0.01), elevated blood urea nitrogen (p < 0.01), higher C-reactive protein (p < 0.01), higher D-dimer (p < 0.01), higher lactic acid (p < 0.01), and higher Quick SOFA (qSOFA), SOFA, TIMI, and GRACE (all scores p < 0.01). On univariate cox regression, troponin elevation (hazard ratio (HR) = 1.85, 95% confidence interval (CI) = 1.18-2.88, p < 0.01), TIMI score >3 (HRv = 1.79, 95% CI = 1.11-2.75, p = 0.01), and GRACE score >140 (HR = 2.27, 95% CI = 1.45-3.55, p < 0.01) were highly associated with mortality, whereas cardiovascular disease (HR = 1.40, 95% CI = 0.89-2.21, p = 0.129) and cardiovascular risk factors (HR = 1.15, 95% CI = 0.73-1.81, p = 0.52) were not. After adjusting for age, use of a non-rebreather or high-flow nasal cannula, hemoglobin <8.5 g/dL, suspected or confirmed source of infection, and qSOFA and SOFA scores (HR = 1.18, 95% CI = 1.07-1.29, p < 0.01) were independently associated with mortality, whereas troponin (HR = 1.08, 95% CI = 0.63-1.85, p = 0.76), TIMI score (HR = 1.02, 95% CI = 0.99-1.06, p = 0.12) and GRACE scores (HR = 1.01, 95% CI = 0.99-1.02, p = 0.10) were not associated with mortality.

Conclusions

Our study shows that troponin, GRACE score, and TIMI score are not independent predictors of mortality in COVID-19 myocardial injury. This may be because troponin elevation in COVID-19 patients may be related to demand ischemia rather than acute coronary syndrome-related. This was shown by the association of troponin with a higher degree of systemic inflammation and end-organ dysfunction. Therefore, we recommend SOFA scores in risk-stratifying COVID-19 patients with myocardial injury.

## Introduction

Coronavirus disease 2019 (COVID-19) is a pandemic caused by a novel strain of coronavirus called severe acute respiratory syndrome coronavirus 2 (SARS-CoV-2). Several studies have shown that myocardial injury is commonly seen in COVID-19 and is associated with a worse prognosis [1]. Although the exact mechanism is still under research, different mechanisms, such as supply-demand mismatch, microvascular thrombi formation, direct viral damage to myocytes, plaque destabilization due to inflammation, and cytokine storm, have been proposed [2,3]. Several retrospective cohort studies have been published on the prevalence and risk factors of troponin elevation in COVID-19 patients. The prevalence of troponin elevation ranges from 7% to 62%, depending upon the severity of COVID-19 [1,4-6]. Studies have shown that the degree of troponin elevation and the presence of cardiovascular disease and cardiovascular risk factors correlate with mortality in COVID-19 patients. The mortality rates among those with elevated troponin range from 20% to 61% [7-9].

However, studies have reported conflicting data on whether troponin is independently associated with mortality [7-9]. The Global Registry of Acute Coronary Events (GRACE) risk score and Thrombolysis in Myocardial Infarction (TIMI) scores predict mortality in patients with myocardial infarction, and the Sequential Organ Failure Assessment (SOFA) score predicts mortality in sepsis. However, the utility of these scores has not been studied in COVID-19-related myocardial injury. Therefore, our primary aim is to determine the independent predictors of mortality in COVID-19-related myocardial injury by comparing the differences in clinical profiles, laboratory values, and special scores of COVID-19 patients with and without troponin elevation. Our secondary aim is to study the predictive value of troponin, GRACE scores, TIMI scores, and SOFA scores on mortality through survival analysis.

This article was previously presented as a meeting abstract at the 2021 AHA Annual Scientific Meeting on November 8, 2021.

## Materials and methods

The electronic medical records of adult patients who were symptomatic for COVID-19 and tested positive for COVID-19 (SARS-CoV-2 reverse transcription polymerase chain reaction positive) from two centers from March 1, 2020, to December 31, 2020, were reviewed. A high-sensitivity troponin-I assay was used to divide patients into two categories. Patients who had troponin levels more than the upper limit of normal (troponin >ULN) were compared with patients who had troponin levels less than the upper limit of normal (troponin <ULN). The upper limit of normal corresponds to the 99th percentile of normal values for troponin. Demographics, clinical features, comorbidities, confounding factors for elevated troponin, vital signs, physical examination findings, laboratory values, troponin trends, electrocardiogram (EKG) changes, COVID-19 treatment, and special scores were collected on the day of troponin elevation. Some laboratory variables such as D-dimer, C-reactive protein (CRP), ferritin, and lactate dehydrogenase (LDH) were collected within seven days of the troponin test as they were not available on the day of troponin elevation. Data were extracted manually by trained investigators through standardized data collection manual. The institutional review board (IRB) of the appropriate institutions approved the study. The IRB waived informed consent due to the retrospective nature of the study.

Statistical analysis

Continuous data were reported as the median and interquartile range, and categorical data were reported as counts and percentages. Wilcoxon rank-sum test was used to analyze continuous data. The chi-square test was used to analyze categorical data. Survival analysis was done with the length of stay as the time variable and death or discharge from the hospital as right censoring. Survival graphs were reported as Kaplan-Meier survivor function with the log-rank test. We compared the clinical profiles, laboratory values, and special scores among patients who had troponin levels more than the upper limit of normal (troponin >ULN) with patients who had troponin levels less than the upper limit of normal (troponin <ULN). We initially ran a univariate cox regression to determine the predictors of mortality with elevated troponin. Variables that were significant in univariate analysis were incorporated in multivariate cox regression. We ran four separate models for troponin, TIMI score, GRACE score, and SOFA score to determine if these variables independently predicted mortality. Kaplan-Meier survival graphs were plotted for key variables with the log-rank test. Any variable with missing data of 10% or less was not included in the analysis. A p-value of less than or equal to 0.05 was considered significant. All data were analyzed with Stata Statistical Software: Release 17 (StataCorp LLC, College Station, TX, USA).

## Results

Clinical features and comorbidities

After screening 1,500 COVID-19 patients, 217 patients who had troponin levels measured during any time of the hospital stay were included. Out of the 217 patients, 85 had elevated troponin, and 132 had normal troponin. Most patients had troponin levels measured during the first week of COVID-19 illness (median = 4, IQR = 1-8). The mean age was 63 (IQR = 50-77) years in the normal troponin group and 79 (IQR = 63-85) years in the elevated troponin group, and the difference was statistically significant (p < 0.01). Cardiovascular diseases (p < 0.01) were associated with elevated troponin but not cardiovascular risk factors (p = 0.887). Patients with elevated troponins also had an increased likelihood of having a mean arterial pressure <65 mmHg (p = 0.01), increased respiratory rate (p = 0.04), increased oxygen support (p < 0.01), and rales on physical examination (p < 0.01) (Appendices).

Laboratory values

Patients with elevated troponin levels had lower absolute lymphocytes (p < 0.01), lower albumin (p < 0.01), higher blood urea nitrogen (p < 0.01), higher creatinine (p < 0.01), higher aspartate aminotransferase (p < 0.01), higher total bilirubin (p < 0.01), higher CRP (p < 0.01), lower LDH (p = 0.01), higher D-dimer (p < 0.01), and higher lactic acid (p < 0.01). Serial troponins were not available for 20% of patients. However, a delta change of 30% (positive or negative change) was more prevalent in patients with elevated troponin (p < 0.01). EKG changes such as tachyarrhythmia (p < 0.01), ST elevation (p < 0.01), ST depression (p = 0.01), and left ventricular hypertrophy (p = 0.02) were more prevalent in patients with elevated troponins (Appendices).

Hospital course and outcomes

In-hospital complications such as atrial fibrillation with rapid ventricular response rates (p < 0.01), congestive heart failure (p < 0.01), chronic obstructive pulmonary disease exacerbation (p < 0.01), intensive care unit (ICU) admission (p < 0.01), suspected or confirmed source of a bacterial infection (p < 0.01), intubation (p = 0.01), vasopressor use (p < 0.01), and death (p < 0.01) were more common in patients with elevated troponin. Higher Systemic Inflammatory Response System (SIRS), Quick SOFA (qSOFA), SOFA, TIMI, and GRACE scores were highly associated with elevated troponin levels (p < 0.01) (Appendices).

Survival analysis

Mortality was 26.5% in the normal troponin group and 54.6% in the elevated troponin group. Patients were categorized into three groups: troponin <ULN, troponin 1-3 times ULN, and troponin >3 ULN. The mortality in each group was 23.6%, 55.2%, and 59.6%, respectively. In unadjusted Cox regression, age (HR = 1.02, 95% CI = 1.00-1.04, p < 0.01), cardiomyopathy (HR = 2.23, 95% CI = 1.10-4.53, p = 0.02), and chronic kidney disease (HR = 1.38, 95% CI = 1.15-1.65) were statistically significant for mortality. Vitals signs such as heart rate >100 (HR = 1.82, 95% CI = 1.14-2.89), respiratory rate (HR = 1.03, 95% CI = 1.00-1.06), and the use of a non-rebreather or high-flow nasal cannula (HR = 1.72, 95% CI = 1.10-2.69) were associated with mortality. Among the laboratory values, hemoglobin <8.5 g/dL (HR = 3.04, 95% CI = 1.38-6.68, p < 0.01), anion gap >12 (HR = 1.66, 95% CI = 1.06-2.6, p = 0.02), serum albumin <3.1 g/dL (HR = 1.82, 95% CI = 1.17-2.84, p < 0.01), blood urea nitrogen (BUN) (HR = 1.00, 95% CI = 1.00-1.01, p = 0.04), tachyarrhythmia (HR = 2.32, 95% CI = 1.48-3.63, p < 0.01), and troponin elevation more than the upper limit of normal (HR = 1.85, 95% CI = 1.18-2.88, p < 0.01) were associated with mortality (Table 1).

**Table 1:** Univariate Cox regression for key variables. ICU: intensive care unit; SOFA: Sequential Organ Failure Assessment; qSOFA: Quick SOFA; TIMI: Thrombolysis in Myocardial Infarction; GRACE: Global Registry of Acute Coronary Events

Variable	Hazard ratio (95% confidence interval)	P-value
Age	1.02 (1.00-1.04)	0.003
Race		
White	Reference	
Black	1.13 (0.63-2.02)	0.67
Hispanic	0.43 (0.20-0.90)	0.026
Asian	1.34 (052-3.46)	0.542
Comorbidities		
Myocardial infarction	1.59 (0.88-2.89)	0.121
Cardiomyopathy	2.23 (1.10-4.53)	0.026
Diabetes mellitus	1.15 (0.73-1.81)	0.529
Hypertension	1.28 (0.76-2.15)	0.344
Statin use	0.95 (0.61-1.47)	0.825
Chronic kidney disease	1.38 (1.15-1.65)	0.000
Hemodialysis or continuous renal replacement therapy	167 (0.85-3.26)	0.132
Vitals		
Mean arterial pressure <65 mm/Hg	1.86 (0.85-4.05)	0.118
Heart rate >100/minute	1.82 (1.14-2.89)	0.011
Respiratory rate	1.03 (1.00-1.06)	0.017
Nonrebreather or high-flow nasal cannula	1.72 (1.10-2.69)	0.016
Labs		
Hemoglobin <8.5 g/dL	3.04 (1.38-6.68)	0.005
Absolute lymphocytes <1 × 10^9^/L	1.14 (0.73-1.78)	0.562
Serum sodium >145 meq/L	1.44 (0.62-3.34)	0.392
Anion gap >12	1.66 (1.06-2.6)	0.024
Serum albumin <3.1 g/dL	1.82 (1.17-2.84)	0.007
Blood urea nitrogen	1.00 (1.00-1.01)	0.04
Creatinine >1.2 mg/dL	1.42 (0.91-2.23)	0.116
Acute kidney injury	1.51 (0.92-2.47)	0.1
Total bilirubin >1.2 mg/dL	1.40 (0.77-2.54)	0.268
Estimated glomerular filtration rate <60 mm/hour	1.31 (0.83-2.06)	0.233
D-dimer	1.00 (0.99-1.00)	0.288
C-reactive protein >100 mg/dL	1.31 (0.83-2.09)	0.239
Electocardiogram changes		
Tachyarrhythmia	2.32 (1.48-3.63)	0.000
ST elevation	1.89 (0.91-3.95)	0.088
ST depression	0.61 (0.15-2.52)	0.503
Left ventricular hypertrophy	1.10 (0.44-2.75)	0.835
Troponin >ULN (upper limit of normal)	1.85 (1.18-2.88)	0.007
Delta change	1.07 (0.67-1.69)	0.766
Medications		
Antibiotics	1.69 (0.81-3.52)	0.162
Steroids	0.54 (0.34-0.85)	0.008
Remdesivir	0.61 (0.33-1.14)	0.123
Tocilizumab	0.62 (0.34-1.12)	0.113
Convalescent plasma	0.53 (0.27-1.03)	0.064
Full-dose anticoagulation	0.85 (0.54-1.33)	0.483
Prophylactic anticoagulation	1.21 (0.77-1.90)	0.404
Antiplatelets	1.73 (1.10-2.72)	0.016
ICU	1.80 (1.11-2.91)	0.016
Intubation	1.45 (0.92-2.27)	0.106
Vasopressor	1.53 (0.96-2.46)	0.073
Scores		
Suspected or confirmed infection	2.7 (1.71-4.25)	0.000
SIRS features	1.27 (1.04-1.55)	0.017
qSOFA >1	2.12 (1.29-3.51)	0.003
SOFA >4	2.29 (1.46-3.57)	0.000
TIMI score >3	1.79 (1.11-2.75)	0.016
GRACE >140	2.27 (1.45-3.55)	0.000

**Figure 1:**
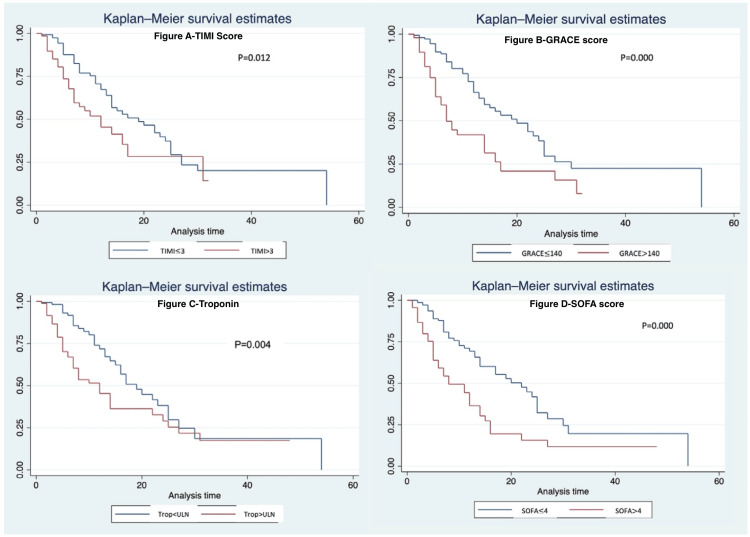
Kaplan-Meier survival analysis for TIMI score >3 (A), GRACE score >140 (B), Troponin >ULN (C), and SOFA score >4 (D). The p-value in the figures represents the p-value in the log-rank test. The x-axis represents the cumulative patients at risk expressed as a fraction, and the y-axis represents the length of stay in days. ULN: upper limit of normal; SOFA: Sequential Organ Failure Assessment; TIMI: Thrombolysis in Myocardial Infarction; GRACE: Global Registry of Acute Coronary Events

The use of antiplatelet (HR = 1.73, 95% CI = 1.10-2.72, p = 0.01) and corticosteroids (HR = 0.54, 95% CI = 0.34-0.85, p < 0.01) were associated with lower mortality. The presence of a confirmed or suspected bacterial infection (HR = 2.7, 95% CI = 1.71-4.25, p < 0.01) and ICU stay (HR = 1.80, 95% CI = 1.11-2.91, p = 0.01) were associated with increased mortality. The presence of systemic inflammatory response syndrome features (HR = 1.27, 95% CI = 1.04-1.55, p = 0.01), qSOFA score >1 (HR = 2.12, 95% CI = 1.29-3.51, p < 0.01), SOFA >4 (HR = 2.29, 95% CI = 1.46-3.57, p < 0.01), TIMI score >3 (HR = 1.79, 95% CI = 1.11-2.75, p = 0.01), GRACE >140 (HR = 2.27, 95% CI = 1.45-3.55, p < 0.01) were all highly associated with mortality. The Kaplan-Meier survival curves plotted for troponin, GRACE, TIMI, and SOFA scores were significant based on the log-rank test (Figure 1).

When combined, cardiovascular disease (HR = 1.40, 95% CI = 0.89-2.21, p = 0.129) and cardiovascular risk factors (HR = 1.15, 95% CI = 0.73-1.81, p = 0.52) were not associated with mortality. We created four separate multivariate Cox regression models for troponin, GRACE score, and TIMI score while adjusting for age, non-rebreather or high-flow nasal cannula, hemoglobin <8.5 g/dL, suspected or confirmed source of infection, and SOFA score. Troponin elevation (HR = 0.79, 95% CI = 0.46-1.34, p = 0.392), TIMI score (HR = 1.03, 95% CI = 0.99-1.07, p = 0.078), and GRACE score (HR = 1.01, 95% CI = 0.99-1.02, p = 0.108) were not independently associated with mortality. SOFA score (HR = 1.18, 95% CI = 1.07-1.29) was strongly associated with mortality in all models (Tables 2-4).

**Table 2:** Multivariate Cox regression model for troponin. SOFA: Sequential Organ Failure Assessment

Variable	Hazard ratio (95% confidence interval)	P-value
Age	1.04 (1.01-1.06)	0.000
High-flow or non-rebreather use	1.80 (1.07-3.03)	0.025
Hemoglobin <8.5 g/dL	5.17 (1.89-14.12)	0.001
Suspected or confirmed source of bacterial infection	2.72 (1.66-4.46)	0.000
SOFA score	1.18 (1.07-1.29)	0.000
Troponin	0.79 (0.46-1.34)	0.392

**Table 3:** Multivariate cox regression model for TIMI score. SOFA: Sequential Organ Failure Assessment; TIMI: Thrombolysis in Myocardial Infarction

Variable	Hazard ratio (95% confidence interval)	P-value
Age	1.03 (1.01-1.05)	0.001
High-flow or non-rebreather use	1.83 (1.08-3.11)	0.024
Hemoglobin <8.5 g/dL	4.95 (1.80-13.6)	0.002
Suspected or confirmed source of bacterial infection	2.81 (1.72-4.61)	0.000
SOFA score	1.17 (1.07-1.27)	0.000
TIMI score	1.03 (0.99-1.07)	0.078

**Table 4:** Multivariate cox regression model for GRACE score. SOFA: Sequential Organ Failure Assessment; GRACE: Global Registry of Acute Coronary Events

Variable	Hazard ratio (95% confidence interval)	P-value
Age	1.01 (0.97-1.05)	0.001
High-flow or non-rebreather use	1.81 (1.07-3.07)	0.024
Hemoglobin <8.5 g/dL	4.79 (1.72-13.3)	0.002
Suspected or confirmed source of bacterial infection	2.62 (1.61-4.25)	0.000
SOFA score	1.11 (1.01-1.23)	0.000
GRACE score	1.01 (0.99-1.02)	0.108

## Discussion

Similar to prior literature, patients with troponin elevation were older and more likely to have cardiovascular diseases such as coronary artery disease, heart failure, cardiomyopathy, stroke, and atrial fibrillation. Patients with elevated troponin also had a higher prevalence of cardiovascular risk factors such as hypertension, diabetes, dyslipidemia, smoking history, chronic kidney disease, and hemodialysis or continuous renal replacement therapy [1,4-6,10]. In addition, the association of hypotension, tachypnea, increased oxygen requirement, lymphopenia, anion gap acidosis, lower albumin, higher BUN and creatinine, and higher inflammatory markers (D-dimer, CRP, LDH) indicates the presence of a higher degree of sepsis and organ dysfunction in patients with elevated troponin when compared with patients with normal troponin. Moreover, the SIRS, qSOFA, and SOFA scores were highly associated with elevated troponin.

There is conflict in current data on whether cardiovascular diseases and cardiovascular risk factors are independently associated with mortality [7-9]. In our cohort, cardiovascular diseases and cardiovascular risk factors were not associated with mortality. In addition, studies are conflicting on whether troponin elevation is independently associated with mortality. One study showed that troponin elevation is independently associated with mortality only in patients with echocardiographic abnormalities [7]. Given that troponin elevation is not independently associated with mortality, it is unclear if obtaining routine troponin levels would change the management of COVID-19 patients. Our study shows that age, hemoglobin <8.5 g/dL, high oxygenation requirements, SOFA scores, and suspected or confirmed source of bacterial infection independently predicted mortality in COVID-19-related myocardial injury. Hence, these variables can be used for risk stratification of COVID-19 patients with elevated troponin.

It is important to distinguish type 1 from type 2 myocardial injury in COVID-19 patients as management is different [11]. Type 1 myocardial injury is due to acute plaque rupture causing coronary artery thrombosis. Type 2 myocardial injury is due to a supply-demand mismatch with or without pre-existing atherosclerosis. For COVID-19 patients suspected of type 1 myocardial injury, an early invasive strategy with dual antiplatelet therapy, statin, beta-blocker, and anticoagulation may be warranted. However, when a COVID-19 patient is suspected of having type 2 myocardial injury, treatment involves supportive care and management of sepsis. Although there is no definitive way to distinguish between the two subtypes other than cardiac catheterization, echocardiographic assessment to study wall motion abnormalities may be useful, as shown in this study [7]. As it is not possible to subject all COVID-19 patients with myocardial injury to cardiac catheterization, we hypothesize that in addition to clinical presentation and echocardiography, the SOFA score may be used to differentiate type 1 from type 2 myocardial injury.

The SOFA score is the gold standard for predicting sepsis mortality [12]. The SOFA score involves seven variables to assess organ function. These include PaO_2_/FiO_2_ ratio, mechanical ventilation, thrombocytopenia, Glasgow Coma Scale score, mean arterial pressure, use of vasopressors, and creatinine and bilirubin levels [12]. This encompasses respiratory, hematological, neurological, cardiovascular, renal, and hepatic markers of sepsis. TIMI and GRACE scores are considered the gold standard to predict mortality in type 1 myocardial injury [13,14]. The TIMI score involves age, the number of cardiovascular risk factors, known coronary artery disease, recent aspirin use, recent severe angina, EKG changes, and troponin elevation. In addition to age, ST deviation on EKG, and elevated troponin, the GRACE score also involves heart rate, systolic blood pressure, creatinine, cardiac arrest, and Killip class [14].

In our study, SOFA scores were independently associated with mortality in COVID-19-related myocardial injury, whereas GRACE and TIMI scores were not independently associated with mortality. This is likely due to the possibility of most patients having type 2 myocardial injury in COVID-19. Although we did not perform cardiac catheterization, it can be safely inferred that troponin elevation in COVID-19 was mainly due to demand ischemia from sepsis rather than acute coronary thrombosis in our cohort. In such patients, management should focus on treating COVID-19 sepsis, not myocardial injury. Patients with low SOFA scores and a higher degree of troponin elevation may have a true type 1 myocardial injury, and these patients may need early catheterization and management for acute coronary syndrome. However, we need more studies comparing type 1 and 2 myocardial injury to make this inference. Limitations of our study include a dual-center design, a small sample size, and the lack of cardiac catheterization data. Our study is one of the few studies which has evaluated the role of GRACE, SOFA, and TIMI scores in COVID-19-associated myocardial injury.

## Conclusions

Our study shows that the SOFA score independently predicts mortality, whereas troponin, GRACE, and TIMI scores do not independently predict mortality in COVID-19-associated myocardial injury. This association suggests the possibility of a preponderance of type 2 myocardial injury in COVID-19 instead of type 1 myocardial injury. Troponin is a cardiac enzyme, and it can be elevated in a variety of disorders, especially severe sepsis. Patients with elevated troponin had higher crude mortality rates. However, after adjusting for markers of sepsis, troponin elevation was not independently associated with elevated mortality. Therefore, we recommend the SOFA score in risk-stratifying COVID-19 patients. The role of the SOFA score in differentiating COVID-19-associated type 1 and 2 myocardial injury is an area of future research.

## Appendices

History and physical examination of troponin elevation in COVID-19

Patients with elevated troponin had a lower BMI (p = 0.03). Fewer Hispanics had troponin elevation when compared with whites (32% of Hispanics with normal troponin and 7.9% with elevated troponin, p < 0.01). None of the clinical features of COVID-19, such as myalgia, fever, cough, sputum, dyspnea, chest pain, and pleuritic chest pain, were different between the two groups.

Patients with elevated troponin had an increased likelihood of having a history of myocardial infarction (p < 0.01), congestive heart failure (p < 0.01), atrial fibrillation (p < 0.01), cardiomyopathy (p < 0.01), diabetes mellitus (p = 0.02), hypertension (p < 0.01), dyslipidemia (p = 0.02), chronic kidney disease (p < 0.01), smoking (p < 0.01), recent surgery (p = 0.01), hematologic malignancy (p < 0.01), and continuous renal replacement therapy or hemodialysis (p = 0.01) when compared to patients who had normal troponin. In summary, cardiovascular diseases (p < 0.01) were associated with elevated troponin but not cardiovascular risk factors (p = 0.887).

Patients with elevated troponin also had an increased likelihood of having a mean arterial pressure <65 mmHg (p = 0.01), increased respiratory rate (p = 0.04), increased oxygen support (p < 0.01), and rales on physical examination (p < 0.01). Other vital signs were not different between both groups. Rare causes for elevated troponin were studied to exclude significant confounding. This includes current burns, physical exertion, recent ablation, pacemaker firing, cardioversion/implantable cardioverter-defibrillator shocks, open heart surgery, heart biopsy, closure of atrial septal defects, biotin use, infectious mononucleosis, and trauma. These variables were mostly absent in our cohort.

**Table 5:** History and physical examination of COVID-19 myocardial injury patients. COVID-19: coronavirus disease 2019; ULN: upper limit of normal; COPD: chronic obstructive pulmonary disease; ICD: implantable cardioverter-defibrillator; ASD: atrial septal defect

Variable	Normal troponin	Elevated troponin (troponin >ULN)	P-value
	N = 132	N = 85	
Age	63 (50-77)	79 (63-85)	0.000
Male sex	70 (53.4%)	45 (52.8%)	0.935
Body mass index	29.1 (25-35.5)	27 (23.5-32.2)	0.033
Race			
White	42 (40.7%)	38 (60.3%)	
Black	19 (18.4%)	15 (25.3%)	0.86
Hispanic	33 (32%)	5 (7.9%)	0.001
Asian	9 (8.7%)	4 (6.3%)	0.268
Duration of COVID-19 illness	4 (1-8)	3 (1-7)	0.417
Clinical symptoms			
Myalgia	34 (26.1%)	26 (31.7%)	0.433
Fever	66 (50%)	40 (47%)	0.577
Cough	77 (58.3%)	40 (48.1%)	0.109
Sputum	14 (10.6%)	11 (13.1%)	0.627
Dyspnea	87 (66.4%)	57 (67%)	0.922
Chest pain	29 (22.1%)	9 (10.7%)	0.066
Pleuritic chest pain	7 (5.3%)	1 (1.1%)	0.111
Comorbidities			
Myocardial infarction	12 (9%)	20 (23.5%)	0.001
Congestive heart failure	14 (10.6%)	24 (27.9%)	0.001
Atrial fibrillation	13 (9.8%)	26 (30.2%)	0.000
Cardiomyopathy	4 (3%)	12 (13.9%)	0.003
Stroke	10 (7.5%)	7 (8.1%)	0.911
Diabetes mellitus	50 (37.8%)	32 (37.6%)	0.02
Hypertension	74 (56%)	67 (77.9%)	0.001
Dyslipidemia	55 (41.6%)	48 (55.8%)	0.029
COPD	13 (9.8%)	10 (11.6%)	0.712
Pulmonary hypertension	2 (1.5%)	2 (2.3%)	0.669
Solid cancer	14 (10.6%)	10 (11.4%)	0.852
Hematologic cancer	0	6 (6.8%)	0.002
Chemotherapy for cancer	3 (2.2%)	3 (3.4%)	0.609
Chronic kidney disease	23 (17.4%)	22 (25.8%)	0.005
History of organ transplant	1 (0.7%)	0	0.414
Liver disease	4 (3%)	1 (1.1%)	0.358
Smoking	31 (23.4%)	7 (8.1%)	0.009
Alcohol abuse	13 (9.8%)	9 (10.4%)	0.619
Recent surgery	4 (3%)	10 (11.7%)	0.012
Hemodialysis or continuous renal replacement therapy	3 (2.2%)	9 (10.4%)	0.011
Any cardiovascular disease	33 (25%)	49 (56.9%)	0.000
Any cardiovascular risk factors	50 (37.8%)	32 (37.2%)	0.887
Rare causes of elevated troponin			
Current burns	0	0	
Physical exertion	0	2 (2.3%)	0.08
Recent ablation	1 (0.7%)	0	0.414
Pacemaker	4 (3%)	5 (5.8%)	0.328
Cardioversion/ICD shocks	0	0	
Heart biopsy/open heart surgery/closure of ASD	0	0	
Biotin use	0	1 (1.1%)	0.219
Infectious mononucleosis	0	2 (2.2%)	0.081
Trauma	1 (0.76%)	1 (1.1%)	0.77
Physical examination			
Systolic blood pressure (mm/Hg)	128 (114-140)	123 (111-150)	0.789
Diastolic blood pressure (mm/Hg)	73 (64-80)	70 (60-81)	0.552
Mean arterial pressure <65 mm/Hg	2 (1.5%)	7 (8%)	0.018
Maximum temperature (F)	98.9 (98.3-100)	98.8 (98.1-100.2)	0.684
Heart rate (beats/minute)	90 (80-100)	92 (80-109)	0.074
Respiratory rate (per minute)	20 (18-24)	22 (19-28)	0.04
Oxygen saturation (%)	96 (94-97)	95 (93-98)	0.615
Oxygenation device			
Room air	93 (70.4%)	45 (52.3%)	0.006
Nasal cannula	12 (9%)	20 (23.2%)	
High-flow nasal cannula	21 (15.9%)	8 (9.3%)	
Jugular venous distension	2 (1.5%)	0	0.246
Rales	15 (11.4%)	22 (25.8%)	0.007

**Table 6:** Laboratory values and outcomes in COVID-19-associated myocardial injury. COVID-19: coronavirus disease 2019; ULN: upper limit of normal; CRP: C-reactive protein; LDH: lactate dehydrogenase; EKG: electrocardiogram; COPD: chronic obstructive pulmonary disease; ICU: intensive care unit; MI: myocardial injury; SIRS: Systemic Inflammatory Response System; SOFA: Sequential Organ Failure Assessment; qSOFA: Quick SOFA; TIMI: Thrombolysis in Myocardial Infarction; GRACE: Global Registry of Acute Coronary Events

Variable	Normal troponin	Elevated troponin (troponin >ULN)	P-value
	N = 132	N = 85	
Laboratory values			
Total white count (10^9^/L)	7.05 (5.36-9.55)	7.8 (5.8-11.5)	0.1
Hemoglobin (g/dL)	12.8 (11.2-14.4)	12.2 (10.8-13.8)	0.127
Platelets (10^9^/L)	207 (151-273)	183 (145-241)	0.11
Bands	0 (0-0)	0 (0-0)	0.37
Absolute neutrophils (10^9^/L)	6.1 (3.8-11.6)	6.3 (4.3-10.7)	0.942
Absolute lymphocytes (10^9^/L)	1.1 (0.7-3.4)	0.7 (0.5-1.2)	0.000
Serum sodium (mmol/L)	135 (133-138)	137 (133-141)	0.005
Serum potassium (mEq/L)	4 (3.7-4.3)	4.1 (3.8-4.6)	0.03
Serum calcium (mg/dL)	8.4 (8.1-8.8)	8.5 (7.8-8.8)	0.795
Blood glucose (mg/dL)	115 (102-159)	139 (107-174)	0.067
Anion gap	9 (7-12)	11 (9-14)	0.000
Serum magnesium (mg/dL)	1.9 (1.5-2.1)	2 (1.7-2.2)	0.058
Serum chloride (mEq/L)	101 (97-104)	102 (100-108)	0.008
Albumin (g/dL)	3.4 (3.1-3.8)	3.1 (2.6-3.6)	0.000
Blood urea nitrogen (mg/dL)	16.5 (11-270	34.5 (18-54)	0.000
Serum creatinine (mg/dL)	0.96 (0.78-1.41)	1.49 (1-2.6)	0.000
Acute kidney injury	84 (64%)	48 (55%)	0.185
Alkaline phosphatase (U/L)	77 (62-91)	78 (111-61)	0.547
Aspartate aminotransferase (U/L)	30 (22-54)	46 (32-84)	0.002
Alanine aminotransferase (U/L)	28 (20-42)	28 (19-54)	0.866
Total bilirubin (mg/dL)	0.5 (0.4-0.7)	0.7 (0.5-0.9)	0.002
D-dimer (ng/mL)	610 (336-1,180)	1,040 (572-3,644)	0.008
CRP (mg/L)	79 (30-149)	139 (68-201)	0.002
LDH (U/L)	332 (232-442)	285 (296-509)	0.012
Lactic acid (mmol/L)	1.5 (1-2.1)	1.9 (1.3-3)	0.004
Serial troponin (ng/mL)			
First troponin	0.03 (0.02-0.03)	0.33 (0.14-0.76)	
Second troponin	0.04 (0.03-0.07)	0.40 (0.22-1.85)	
Third troponin	0.06 (0.03-0.13)	0.43 (0.21-1.9)	
EKG changes			
Tachyarrhythmia	15 (11.4%)	36 (44.4%)	0.000
Bradyarrhythmia	2 (3%)	2 (2.4%)	0.654
ST elevation	4 (3%)	8 (9.7%)	0.008
ST depression	1 (0.76%)	6 (7.3%)	0.01
Left ventricular hypertrophy	5 (3.8%)	10 (12.2%)	0.023
PR segment depression	0	2 (2.4%)	0.075
In-hospital complications			
Multifocal pneumonia	84 (63.6%)	50 (59.5%)	0.433
Atrial fibrillation with rapid ventricular rate	9 (6.8%)	16 (19%)	0.000
Pulmonary embolism	1 (0.7%)	1 (1.2%)	0.75
Congestive heart failure	1 (0.7%)	16 (20.5%)	0.000
Acute stroke	0	1 (1.1%)	0.213
COPD exacerbation	2 (1.5%)	1 (1.1%)	0.825
ICU admission	32 (24.2%)	42 (48.8%)	0.000
Intubation	24 (18.1%)	29 (33.7%)	0.011
Vasopressor	12 (9%)	21 (25.3%)	0.002
Death	35 (26.5%)	47 (54.6%)	0.000
Length of stay	6 (2-12)	6 (4-12)	0.223
Treatment			
Antibiotics	86 (65.1%)	64 (75.2%)	0.171
Plaquenil	61 (46.2%)	27 (31.7%)	0.026
Steroids	57 (43.1%)	42 (49.4%)	0.441
Remdesivir	18 (13.6%)	18 (21.1%)	0.164
Tocilizumab	17 (12.8%)	7 (8.2%)	0.266
Convalescent plasma	10 (8%)	8 (10.2%)	0.627
Full-dose anticoagulation	33 (25%)	43 (50%)	0.627
Prophylactic anticoagulation	66 (50%)	42 (49.4%)	0.000
Antiplatelet	29 (21.9%)	41 (47.6%)	0.824
Treated as MI	4 (3%)	17 (19.7%)	0.000
Stress test	1 (0.7%)	0	0.3
Catheterization	0	0	
Sepsis scores			
SIRS	1 (1-2)	2 (1-2)	0.032
qSOFA	1 (1-1)	1 (0-2)	0.000
SOFA	2 (1-3)	4 (2-5)	0.000
Suspected or confirmed source of infection	28 (21.8%)	36 (44.4%)	0.000
MI scores			
TIMI score	1 (0-2)	3 (2-4)	0.000
GRACE score	90 (65-112)	142 (114-156)	0.000
